# The contributions of gender identification and gender ideologies to the purposes of social media use in adolescence

**DOI:** 10.3389/fpsyg.2022.1011951

**Published:** 2023-01-10

**Authors:** Adriana M. Manago, Abigail S. Walsh, Logan L. Barsigian

**Affiliations:** Department of Psychology, University of California, Santa Cruz, CA, United States

**Keywords:** adolescent social media use, gender differences, gender identity, masculinity and femininity ideology, uses and gratifications theory

## Abstract

Gender differences in adolescent social media use are often documented in the research literature, yet few studies delve into why they occur. Accordingly, we investigated whether gender identification and gender ideologies are associated with five major purposes of social media use in adolescence (emotion and activity bonding with friends, social compensation, appearance validation, and bullying). Participants were 309 cisgender U.S. high school students (Mage = 15.74; 59% girls; 53% white) primarily using Instagram and Snapchat but also TikTok (more popular with girls) and Discord (more popular with boys) in 2019. Girls reported greater use of social media for emotion bonding, appearance validation, and social compensation compared to boys, who reported greater competitive activity bonding. Girls and boys did not differ in their use of social media for bullying. In linear regressions, masculinity ideology predicted purposes associated with girls (appearance validation, social compensation), as well as those associated with boys (competitive activity bonding), regardless of gender. Femininity ideology uniquely predicted emotion bonding and social compensation but only mediated the effect of gender for the latter. Findings illustrate that gender is important for understanding uses and gratifications of social media in adolescence, but traditional masculinity ideology is similar across genders and relates to multiple functions of social media in boys’ and girls’ lives. More work is needed to conceptualize gender beliefs and values in Gen Z, given recent challenges to gender binary ideology and low reliability of the scales in this study, which were developed before social media.

## Introduction

Research over the past two decades has illustrated that gender is associated with adolescents’ experiences with interactive digital media (Manago et al., 2008; Ringrose, 2010). Large survey studies in the U.S. and U.K. have found that girls spend more time on their smartphones involved in social networking sites compared to boys, who spend more time playing internet games with friends compared to girls (Rideout et al., 2010; Przybylski and Weinstein, 2017). Whereas time spent on social networking sites is most consistently associated with internalizing issues such as depression, which are often higher in girls (McCrae et al., 2017; Keles et al., 2020), problematic gaming is most frequently associated with externalizing issues such as aggression, which are often higher in boys (Desai et al., 2010; Coyne et al., 2018). Gendered patterns of social media uses and consequences may be assumed to be influenced by cultural norms and expectations (gender roles) but the premise largely goes untested. A lack of clarity regarding origins of gender differences in adolescents’ social media use can give the impression that female–male binaries are natural and inevitable features of human motivation and development in adolescence, evolving in the distant past, and persisting through cultural and technological change (e.g., Tifferet, 2019).

One cultural source of gender differences in social media use may be found in adolescents’ identification with gender ideologies. Gender ideologies are socially shared beliefs, values, and expectations about biological sex and human behavior that form part of the macro-system shaping individual development (Manago and Pacheco, 2019; Rogers et al., 2020; Sung, 2021). Although ideologies are often conceptualized as socialization outcomes of mainstream media use in adolescence (Giaccardi et al., 2016; Anyiwo et al., 2018), they also inform why youth use media in the first place, especially interactive media (Manago et al., 2008; van Oosten et al., 2017). Uses and Gratifications Theory (UGT) posits that individuals’ conscious goals and intentions, socialized in cultural environments, drive their media selection, forms of engagement, and benefits of use (Katz et al., 1973). Although UGT can be constructive for examining how social structures such as those associated with gender, class, and race are translated into disparate motivations, uses, and outcomes of social media, the theory is often employed in ways that essentialize individual differences, particularly with regard to gender (e.g., Kircaburun et al., 2020). In addition, while UGT has shed light on importance of understanding adults’ reasons for using a specific platform or feature (Malik et al., 2016; Phua et al., 2017), less is known about adolescents’ unique developmental needs and goals within the context of their personally customized “polymedia” environments (Madianou and Miller, 2013; Nesi et al., 2018).

To fill these gaps, the current study examined how gender identification and traditional masculinity and femininity ideologies contribute to common purposes of social media use during adolescence: bonding with friends, social compensation, appearance validation, and bullying. Our aims were to go beyond simple frequency of use measures to examine different goals of social media use, both beneficial and harmful (Odgers, 2018; Odgers and Jensen, 2020), and to shed light on cultural-psychological factors involved in individual differences in social media uses (Beyens et al., 2021; Manago and McKenzie, 2022). Instead of targeting a specific platform, we aimed to understand how adolescents integrate multiple platforms and tools in contemporary “polymedia” environments (Madianou and Miller, 2013) and defined social media broadly, as any kind of internet-based medium that enables synchronous or asynchronous social interaction and self-presentation at various scales, from dyads to large groups (Carr and Hayes, 2015). We briefly summarize research on gender differences in adolescents’ uses of social media and then describe how traditional gender ideologies could contribute to those differences.

## Gender differences in adolescents’ uses of social media

### Social media for friendship bonding

Perhaps the most common and beneficial purpose of adolescents’ use of social media is to bond with close friends from their offline lives (Reich et al., 2012; Yau and Reich, 2018). Gender differences in the use of social media to nurture ties with close friends have been found since the early days of social network sites (e.g., Thelwall, 2008). When Facebook became popular, research with U.S. college students found that compared to men, women had more close friends on Facebook and communicated with them more frequently (Pempek et al., 2009; Ellison et al., 2014). Research with adolescents also found that girls used social media more with close friends than did boys (Lenhart and Madden, 2007; Rideout et al., 2010) and engaged in greater self-disclosure to increase friendship closeness (Schouten et al., 2007; Valkenburg et al., 2011).

As platforms multiplied, recent work suggests that gender differences in social media use for friendship bonding depend on the platform under consideration (Vannucci and McCauley Ohannessian, 2019). Girls in the U.S. are more likely to use Instagram and Snapchat with friends compared to boys (Vannucci and McCauley Ohannessian, 2019; McCauley Ohannessian and Vannucci, 2021), who are more likely to use Twitter and discussion boards (Vannucci and McCauley Ohannessian, 2019) or YouTube (Anderson and Jiang, 2018). Additionally, online gaming has also become central to U.S. adolescent boys’ friendships (Lenhart, 2015; Twenge and Martin, 2020), with platforms such as Discord hosting multiplayer online games such as Fortnite alongside affordances for sociality (i.e., chat) at various scales. These online gaming communities have been shown to be conducive to adolescents’ friendship closeness (Mittmann et al., 2022). Developmental research indicates that girls and boys in the U.S. spend equivalent time fostering friendship but girls tend to do so by sharing feelings whereas boys bond through games and sports (Rudolph and Dodson, 2022). To be more inclusive of different kinds of friendship bonding occurring online, we asked adolescents about their use of social media to bond through emotions and to bond through activities.

### Social media for social compensation

Another potentially beneficial purpose is the use of social media to overcome offline barriers to increase social resources—often referred to as “social compensation” (Desjarlais and Willoughby, 2010; Abbas and Mesch, 2018). Unique affordances of digital communication such as accessibility, asynchrony, and anonymity enable some adolescents to overcome obstacles in the physical world such as distance or eye contact to acquire new relationships and social support (Valkenburg and Peter, 2011; Antheunis et al., 2016). The potential for social compensation with technology-mediated communication has fueled debates about whether isolated youth such as lonely, anxious, or minoritized youth have more to gain from social media than their already socially rich counterparts (poor-get-richer/social compensation hypothesis), or whether social media magnify offline disparities in social resources (rich-get-richer/social enhancement hypothesis) (Cheng et al., 2019; Pouwels et al., 2021).

With respect to gender, research primarily with adults has supported the idea that while women use social media to nurture close offline ties, men use social media for social compensation, particularly to expand their social networks (Bonds-Raacke and Raacke, 2010; Muscanell and Guadagno, 2012; Ellison et al., 2014). Studies with adolescents have shown that boys are more interested in finding new friends online compared to girls (Abbas and Mesch, 2018), and that digital communication is particularly useful for shy boys to overcome social anxiety to connect with peers (Desjarlais and Willoughby, 2010). Potential explanations for these gender differences include traditional gender roles differentially related to domestic and public social spheres (Tifferet and Vilnai-Yavetz, 2014) and women’s greater vulnerability and thus concern for privacy compared to men, leading them to restrict their communication to already established close ties (Tifferet, 2019). However, some recent studies have not found gender differences in the use of social media to increase social networks and resources (Shane-Simpson et al., 2018; Quinn and Epstein, 2019). Studies considering multiple platforms find that young men are more likely to expand social resources using Twitter, whereas young women are more likely to do so using Instagram or TikTok (Shane-Simpson et al., 2018; Bossen and Kottasz, 2020).

### Social media for appearance validation

A common but perhaps less salubrious purpose of social media among adolescents is posting attractive photos to receive approbation and public endorsement for one’s value and self-worth. Visual-based platforms such as Instagram seem designed to feed especially into girls’ “post-feminist” desires in the West to establish themselves as valuable to their peer groups through physical appearances that reinforce traditionally dominant standards of sexiness and appeal for femininity (Ringrose and Barajas, 2011; Mascheroni et al., 2015). Indeed, research in the U.S. has shown that adolescent girls invest significant time and effort into posting photos of themselves and monitoring quantified peer approval through “likes” (Yau and Reich, 2018). Although adolescent boys and college men also use social media for appearance validation (Kapidzic and Herring, 2015; van Oosten et al., 2017), adolescent girls report engaging more frequently in behaviors such as self-objectification online compared to boys (Salomon and Brown, 2019) and have more appearance-related consciousness and body shame compared to boys (Choukas-Bradley et al., 2020), suggesting that social media for appearance validation is more impactful in girls’ development.

### Social media for bullying

One of the most worrisome purposes of social media use in adolescence is cyberbullying, defined as a form of traditional bullying (repeated intentional aggression involving power imbalance with known peers) through the use of electronic communication (Olweus and Limber, 2018). Although research on cyberbullying has expanded rapidly, primarily in North America and Europe (Smith and Berkkun, 2017), measurement issues make it difficult to estimate prevalence rates, which range from 1–41% of adolescents in U.S. based studies (Selkie et al., 2016). Although not always consistent, studies tend to find that boys are more likely to cyberbully compared to adolescent girls (Guo, 2016; Shapka et al., 2018). Interestingly, gender differences in cyberbullying perpetration are smaller and less consistent compared to offline bullying (Smith et al., 2019). The anonymity and asynchrony of cyberbullying may mimic indirect forms of aggression (i.e., relational versus physical) found to be greater among girls compared to boys in face-to-face settings (Bowie, 2007). Others have argued that masculine traits may be more important than being male in predicting cyberbullying perpetration (Wright, 2020).

## Do gender ideologies explain gender differences in social media uses?

One limitation of research on gender differences in adolescent social media use is that gender essentialism is baked into binary comparisons that assume two classifications of humans with distinct psychological characteristics, an assumption that has been debunked by psychological meta-analyses (Hyde, 2014) and by neuroscience and behavioral neuroendocrinology (Hyde et al., 2019). Gender differences may be better understood in the context of adolescents’ negotiations of cultural constructions of gender such as masculinity and femininity ideologies that organize their learning (Manago and Pacheco, 2019; Rogers et al., 2020; Sung, 2021). Gender ideologies in the U.S. have long been dominated by widespread and hegemonic cultural beliefs and values that are premised on a gender binary based on sex at birth and that prescribe ideals such as strong and assertive for men and nice and nurturing for women (Tolman and Porche, 2000; Chu et al., 2005). Femininity and masculinity ideologies maintain gender hierarchy on societal and individual levels by diminishing women’s agency in relationships through self-objectification and self-silencing (Tolman and Porche, 2000), while valuing men who are dominating, independent, stoic, promiscuous, and homophobic (Chu et al., 2005). Adolescents contend with these hegemonic gender ideologies, either accommodating or resisting them in their identity development (Rogers et al., 2020).

Because gender ideologies can be transmitted through media, they have often been studied as socialization outcomes in media effects research with adolescents (Giaccardi et al., 2016; Anyiwo et al., 2018). However, a transactional developmental perspective recognizes that adolescents’ developing sense of selves guide their engagement with media, which further shapes their identities (Valkenburg et al., 2016). With the proliferation of social media platforms, adolescents have more choices about where and how to engage socially (Madianou and Miller, 2013). Girls’ accommodation to submissive femininity ideology could drive their use of social media to align with traditional gender beliefs and values, such as the use of social media for appearance validation. Indeed, studies have shown that believing in the importance of appearing sexually attractive for boys—a central component of traditional femininity ideology—motivates girls’ participation in online self-objectification (Ringrose, 2010; Mascheroni et al., 2015; van Oosten et al., 2017). Femininity ideology may also motivate use of social media to align with values for emotional sensitivity and nurturing others. One study with U.S. college students found that femininity ideology was better than gender identification in predicting emotion bonding through emoticons in text messages (Ogletree et al., 2014).

Likewise, boys’ accommodation to traditional masculinity could drive their social media use for competitive activity bonding, bullying, and social compensation insofar as these activities are ways for boys to limit their vulnerability and gain power in their relationships. Traditional masculinity, particularly values for heterosexuality and physical dominance, is a central ideological foundation for bullying in adolescence (Pascoe, 2007; Rosen and Nofziger, 2019), often further perpetuating gender, class, and race power dynamics in peer relations (Ringrose and Renold, 2010; Martinez-Pecino and Durán, 2019). Although there is very little research on peer bonding through online gaming, offline video gaming has been shown to be associated with young men’s dominant masculinity ideology (Gilbert et al., 2018) and their sexist beliefs and values (Stermer and Burkley, 2012; Fox and Potocki, 2016). Boys’ use of social media to assert power through aggression, bond with friends through competitive activities, and to expand their social circles could be driven, in part, by a desire to assert agency and power in their relationships and align with traditional masculinity ideology.

### Current study

Research over the past two decades suggests gender continues to be an important social structure shaping uses and consequences of social media in adolescence. To examine gender as a context in adolescents’ social media use, we tested whether adolescents’ identification with traditional gender ideologies help explain gender differences in the purposes of their social media use. Instead of focusing on a specific platform, we asked adolescents about the degree to which they use their top three social media platforms for the purposes of emotion and competitive activity bonding, social compensation, appearance validation, and bullying. Rather than assuming girls identify with femininity and boys with masculinity, we asked all adolescents to report their level of identification with both masculinity and femininity ideologies in their relationships.

**Research Question:** Are there gender differences in the purposes of adolescents’ social media use and are they mediated by identification with cultural ideologies for gender?

Hypothesis 1 Emotion Bonding:

A) Higher among girls than boys

B) Gender differences mediated by femininity ideology

Hypothesis 2 Competitive Activity Bonding:

A) Higher among boys than girls

B) Gender differences mediated by masculinity ideology

Hypothesis 3 Social Compensation:

A) Higher among boys than girls

B) Gender differences mediated by masculinity ideology

Hypothesis 4 Appearance Validation:

A) Higher among girls than boys

B) Gender differences mediated by femininity ideology

Hypothesis 5 Bullying:

A) Higher among boys than girls

B) Gender differences mediated by masculinity ideology

## Materials and methods

### Participants

The final sample included 309 9-12^th^ grade students enrolled in a public high school in northern California. We asked participants for their sex assigned at birth (female, male, other/write-in) and the gender they identify with now (girl/woman, boy/man, other/write-in). Combining these two questions, 182 participants identified as a cisgender girl/woman and 127 identified as a cisgender boy/man (i.e., sex assigned at birth matches current gender identification). One survey respondent reported a non-binary gender identity; given the limitations of meaningful quantitative analyses with this small a sample size, we removed this participant from the analyses and focused on the ways that traditional gender ideologies shape cisgender adolescents’ use of social media. We also removed two participants who reported zero social media use. In response to an open-ended question about ethnic group background, the majority of the sample identified as White (53%), followed by Latino/a/x (19%). The rest of the sample identified with multiple ethnic/racial groups such as “Latina/White” or “Japanese, Mexican, Italian” (17%), with a few participants identifying as Asian or Pacific Islander (4%), Middle Eastern (2%), and Black/African American (1%). Additionally, 5% of the sample did not provide their ethnic background. There were no gender differences in ethnic group identifications. A majority of participants’ parents had at least a college degree.

### Measures

#### Sociodemographics

In addition to gender and ethnicity, participants were asked their age in years and months and the highest level of education achieved by their mother/female guardian/or parent one and father/male guardian/or parent two on a scale from (1 = *Elementary school* to 10 = *Ph.D., M.D., MBA, Law school grad*). Participants also used a likert scale from (1 = *Not at all/Never* to 5 = *Extremely/Very regularly)* to answer questions about their degree of religiosity, praying, attending services, which we averaged for a total religiosity score (*α* = 0.87).

#### Purposes of social media use

Because research on adolescent social media use focuses on overall frequency of use (Twenge and Martin, 2020), engagement with platform features such as photo-tagging (Dhir and Torsheim, 2016), or specific forms of communication such as self-disclosure (Valkenburg et al., 2011), there are no scales for measuring purposes of adolescents’ social media use. Therefore, our surveys first provided participants with a definition of social media (“websites and applications where users can create and share content and socialize”) along with examples including Facebook, Instagram, Snapchat, Twitter, WhatsApp, Tumblr, TikTok, Reddit, Steam, Discord, and MMORPGs (Massively Multi-player Online Role Playing Games). Participants were then asked to identify the top three social media sites they used most often, either by circling the provided examples or writing their own. Next, participants were asked to think about the reasons they use social media and to rate whether various goals of social media use applied to them on a scale from 1 = *Not at all* to 5 = *Very much so*. We report McDonald’s omega (*ω*) as an estimate of scale reliability because it does not assume that items are tau-generic (Hayes and Coutts, 2020).

**Emotion Bonding.** We constructed three items about the use of social media to bond with close friends through emotions: “I use social media to talk about personal problems with close friends,” “I use social media to share personal thoughts with close friends,” and “I use social media to show emotional support to my close friends.” The scale demonstrated good internal consistency (*ω* = 0.84).

**Competitive Activity Bonding.** We constructed three items about the use of social media to bond with close friends through activities: “I use social media to challenge my close friends to see who is better at something,” “I use social media to joke around with my close friends,” and “I use social media to do fun activities with my close friends.” Reliability for this scale was low but close to a 0.70 cutoff value (*ω* = 0.67).

**Social Compensation.** We constructed three items about the use of social media to expand one’s social resources beyond face-to-face contexts: “I use social media to find new friends,” “I use social media to talk about things that I do not want to talk about face-to-face,” and “I use social media to interact with people who are more like me than the people I know from school.” Reliability for this scale was low but close to a 0.70 cutoff value (*ω* = 0.69).

**Appearance Validation.** We constructed three items about the use of social media to gain status with peers through looks and sex appeal: “I use social media to show “hot” photos of myself,” “I use social media to see what others think about how I look,” and “I use social media to see how my appearance compares to others.” The scale demonstrated good internal consistency (*ω* = 0.83).

**Bullying.** To measure social media use for bullying we adapted an abbreviated 4-item version of the Olweus Bully/Victim Questionnaire scale asking adolescents the extent to which they used social media to cyberbully in the past year from 1 = *Never* to 5 = *Several times a week* (Olweus, 1996). For example, “I spread false rumors about peers on social media and tried to make others dislike them” and “I threatened to hit, kick, push or shove a peer on social media.” Items on this scale were internally reliable (*ω* = 0.77).

#### Gender ideologies

To measure adolescents’ identification with traditional gender ideologies, we adopted previous scales for measuring adolescents’ internalization of beliefs and values associated with dominant masculinity and submissive femininity ideologies in peer relations (Tolman and Porche, 2000; Chu et al., 2005). Schools asked us to limit the length of the surveys distributed to adolescents and so we used abbreviated versions of these scales to comply with time constraints.

Adolescents’ identification with masculinity ideology was measured using an abbreviated 6-item version of the Adolescent Masculinity Ideology in Relationships Scale (AMIRS; Chu et al., 2005). Items on this scale are about being dominant and invulnerable in friendships and dating/sexual relationships, for example, “It’s important to act like I am sexually active and knowledgeable, even if I am not.” For some of the items we needed to reword them to remove reference to gender (e.g., “If a guy tells people his worries, he will look weak” changed to “If I tell people my worries, I would look weak”). All participants were asked to rate how much each item described themselves (1 = *Not at all like me* to 4 = *A lot like me*). McDonald’s omega was low for our 6-item scale (*ω* = 0.60). Nevertheless, items on the scale capture the content of our conceptualization of the identification with hegemonic masculinity ideology and each of the six AMIRS items were highly correlated with overall masculinity ideology, Pearson correlations all greater than 0.48. See factor analysis in Appendix.

Adolescents’ identification with femininity ideology was measured using an abbreviated 6-item version of the subscale in the Adolescent Feminine Ideology Scale (AFIS) that assesses experiences of a submissive self in relationships (Tolman and Porche, 2000). The subscale included items about suppressing authentic selves to avoid conflict such as, “I often change the way I do things in order to please someone.” All participants were asked to rate how much each item described themselves (1 = *Not at all like me* to 4 = *A lot like me*). Our 6-item scale had very low internal consistency (*ω* = 0.50). Removal of three items related to self-expression, “I express my opinions only if I can think of a nice way of doing it,” “I tell my friends what I think even when it is an unpopular idea” (reverse scored), and “I usually tell my friends when they hurt my feelings” (reverse scored) improved omega (*ω* = 0.59) so we computed the mean score for femininity ideology without these items. Although the omega is still below a standard cutoff-point for reliability, items on the scale capture the content of our conceptualization of identification with femininity ideology and each of the remaining three AFIS items were highly correlated with overall femininity ideology, Pearson correlations all greater than 0.70. See factor analysis in Appendix.

### Procedure

The school was recruited as part of outreach to principals and advisors in gender and sexual diversity clubs at a variety of schools. A paper and pencil survey was administered to students during school hours in six different world language classes spanning 9^th^ – 12^th^ grades. Students whose parents had signed consent forms participated in the survey. Participants answered items on ideologies, social media uses, and lastly, sociodemographic measures. Researchers were available to answer participants’ questions while they were taking the survey. The survey took approximately 20 min for participants to complete.

### Data analysis plan

We first conducted a principal component analysis on all the social media purposes items to assess whether variability could be reduced to a smaller set of five components to test our hypotheses. The KMO measure of sampling adequacy of the correlated survey items was 0.78, indicating a factor analysis was appropriate for the data. We chose a principal component analysis with varimax rotation as we expected to derive a reduced set of variables on adolescents’ social media use that would be orthogonal to one another. Based on the decision rule of eigen values greater than one, the analysis retained a five-component solution. Together the five factors accounted for 68% of the variability in the data. The rotated component matrix revealed that the four bullying items loaded clearly onto component one (factor loadings >0.62), the three emotion bonding items loaded clearly onto component two (factor loadings >0.78), the three appearance validation items loaded clearly onto component three (factor loadings >0.76), and the three social compensation items loaded clearly onto component four (factor loadings >0.58). For component five, two activity bonding items clearly loaded onto this factor (factor loadings >0.79) but one item, “I use social media to challenge my close friends to see who is better at something,” loaded onto both component five (activity bonding) and component one (bullying). We retained the item on the activity bonding variable based on our theoretical conceptualizations of competitive activities as a way to connect with friends and bullying as intention to inflict harm on others. See factor analysis in Appendix.

We also examined whether the gender ideology and social media variables had non-normal distributions that would violate assumptions of ANOVA and linear regression. There was significant positive skew and kurtosis in the bullying variable for girls and for boys. We performed square root and log 10 transformations but neither strategy fully normalized the variable. Therefore, non-parametric statistical tests were used when considering bullying. We transformed the bullying variable into two categories: never bullied (response 1 on the scale; *n* = 115) or bullied (responses 2–5; *n* = 194).

To test our hypotheses, we first conducted a MANOVA to examine whether girls and boys differ in their social media use for emotion bonding, competitive activity bonding, social compensation, and appearance validation. Chi-square analysis was used as a non-parametric method for testing gender differences in social media use for bullying. Next, following the steps outlined by Baron and Kenny (1986) for mediation, we used linear regression to test whether gender ideologies accounted for unique variation in the social media purposes and whether the effect of gender would be reduced to zero with gender ideologies included in the models. We also included sociodemographic factors in the regression models if they were associated with gender or the social media purposes. To minimize the accumulation of alpha, we used the Benjamini-Hochberg method to adjust for inflation by rank ordering *p* values associated with our 13 tests of significance (five tests of gender differences in social media purposes plus eight tests of associations between the two gender ideologies and the four social media purposes that differed by gender).

## Results

### Adolescents’ platform preferences

The most popular platforms in adolescents’ top three were Instagram (92% of participants), Snapchat (88% of participants), and TikTok (48% of participants). All platforms that adolescents identified are in Table 1, from most popular to least popular. Table 1 also includes proportions of girls and boys identifying a particular platform and Chi-square significance tests. There were no gender differences in the top two platforms, but girls were more likely than boys to select TikTok and Tumblr in their top three; boys were more likely than girls to select Discord, Reddit, MMORPGs, and Steam. Platform preferences were not associated with gender ideologies, except for girls’ identification with masculinity ideology and the presence of Reddit in their top three (*r* = 0.21, *p* < 0.01).

**Table 1:** Gender differences in adolescents’ top three social media platforms.

Platform	% of Girls’ Top 3 (*n* = 182)	% of Boys’ Top 3 (*n* = 127)	*χ* ^2^
Instagram	93%	89%	1.91
Snapchat	91%	85%	2.30
TikTok	66%	20%	63.49***
Twitter	13%	17%	1.32
Discord	2%	23%	36.51***
Reddit	3%	13%	12.81***
Facebook	6%	7%	0.13
MMORPGs	0.5%	13%	20.89***
Steam	0%	9%	16.35***
YouTube	3%	6%	1.53
Tumblr	3.8%	0%	5.00*
WhatsApp	1.6%	1.6%	0.00

Percentages represent proportions of girls and proportions of boys identifying a particular platform in their top three social media platforms. *p < 0.05. **p < 0.01. ***p < 0.001.

We also explored associations between platforms and social media purposes, controlling for gender. The presence of Instagram in adolescents’ top three platforms was positively associated with using social media for emotion bonding (*r* = 0.16, *p* < 0.01) and for competitive activity bonding (*r* = 0.16, *p* < 0.01). The presence of TikTok was associated with using social media for emotion bonding (*r* = 0.14, *p* < 0.05) and social compensation (*r* = 0.12, *p* < 0.05). Discord (*r* = 0.13, *p* < 0.05) and Steam (*r* = 0.17, *p* < 0.01) were associated with using social media for bullying. Reddit (*r* = −0.12, *p* < 0.05) and Steam (*r* = −0.14, *p* < 0.05) were also negatively associated with using social media for emotion bonding.

### Gender differences in social media purposes

Table 2 presents statistical significance tests of gender differences in adolescents’ purposes of social media use. For both girls and boys, competitive activity bonding was the most strongly endorsed use, followed by emotion bonding, social compensation, and appearance validation. However, girls reported using social media more than boys for emotion bonding, appearance validation, and social compensation. Boys reported using social media more than girls for activity bonding. Gender differences were not found in the dichotomized bullying variable. Hypotheses 1A, 2A, 4A were supported. Hypothesis 3A and 5A were rejected.

**Table 2:** Gender differences in adolescents’ sociodemographic backgrounds, gender ideologies, and purposes of social media use.

Variable	Girls (*n* = 182)		Boys (*n* = 127)		*F*/*χ*^2^
	*M*	*SD*	*M*	*SD*
Sociodemographics
Age	15.62	0.99	15.93	0.97	7.46**
Mother/Parent1 Educ	6.13	2.25	6.50	2.13	2.10
Father/Parent2 Educ	5.90	2.28	6.40	2.33	3.46
Religiosity	2.22	1.09	2.10	1.06	1.10
Gender ideologies
Femininity	2.13	0.63	1.97	0.66	4.57*
Masculinity	1.61	0.42	1.61	0.48	0.001
Social media purposes
Activity bonding	3.18	0.76	3.50	0.87	11.33***
Emotion bonding	2.99	1.08	2.54	0.98	13.60***
Social compensation	2.54	0.98	2.30	0.97	4.85*
Appearance Valid	2.02	0.98	1.51	0.76	23.27***
Bullying	37% Never 63% Yes		38% Never 62% Yes		0.03

Parent Education 1–10 scale; Religiosity 1–5 scale; Gender Ideologies 1–4 scale; Social Media Purposes 1–5 scale, Bullying transformed to dichotomous variable.

*p < 0.05. **p < 0.01. ***p < 0.001.

### Gender ideologies as mediators between gender and social media purposes

As displayed in Table 2, only identification with femininity ideology differed by gender. Femininity and masculinity ideologies were positively correlated for girls (*r* = 0.32, *p* < 0.001) but unrelated for boys (*r* = 0.16, *p* = 0.08). To examine whether femininity and masculinity ideologies statistically predicted social media purposes after accounting for gender, we ran four regression models with each of the social media purposes that differed by gender as outcomes (all but bullying), controlling for age (associated with gender), and religiosity (associated with emotion bonding and competitive activity bonding). Parents’ education was not associated with any variables of interest and therefore not included in the models. Variance inflation factors were close to 1 for all variables, indicating multicollinearity was not an issue. Statistics for the regression equations are summarized in Tables 3–6.

**Table 3:** Linear multiple regression predicting social media for emotion bonding.

	*b*	CI_95%_ for *b*		*β*	*r*	*sr* ^2^
		Lower	Upper		
Predictor variables
Age	0.07	−0.05	0.20	0.07	0.03	0.07
Gender	0.48	0.23	0.73	0.22**	0.22**	0.22
Religiosity	−0.11	−0.22	0.00	−0.11	−0.11	−0.11
Femininity ideology	0.25	0.05	0.44	0.15*	0.18*	0.14
Masculinity ideology	−0.02	−0.30	0.26	−0.01	0.02	−0.01

*R*^2^ = 0.09, Adjusted *R*^2^ = 0.07, *F*(5, 282) = 5.40, *p* < 0.001.

*b*, Unstandardized coefficient; CI, Confidence interval; *ß*, Standardized coefficient; *r*, Zero-order correlation; *sr*^2^, Squared part correlation. *p < 0.02. **p < 0.001.

**Table 4:** Linear multiple regression predicting social media for competitive activity bonding.

	*b*	CI_95%_ for *b*		*β*	*r*	*sr* ^2^
		Lower	Upper			
Predictor variables
Age	0.06	−0.04	0.15	0.07	0.11	0.07
Gender	−0.29	−0.48	−0.10	−0.17*	−0.17*	−0.17
Religiosity	−0.09	−0.17	−0.01	−0.12	−0.12	−0.12
Femininity ideology	0.18	0.04	0.33	0.15*	0.18*	0.14
Masculinity ideology	0.35	0.14	0.56	0.19*	0.22**	0.19

*R^2^* = 0.12, Adjusted *R*^2^ = 0.11, *F*(5, 282) = 7.74, *p* < 0.001.

*b*, Unstandardized coefficient; CI, Confidence interval; *ß*, Standardized coefficient; *r*, Zero-order correlation; *sr*^2^, Squared part correlation. *p < 0.02. **p < 0.001.

**Table 5:** Linear multiple regression predicting social media for social compensation.

	*b*	CI_95%_ for *b*		*β*	*r*	*sr* ^2^
		Lower	Upper			
Predictor variables
Age	−0.06	−0.18	0.05	−0.06	−0.07	−0.06
Gender	0.20	−0.03	0.43	0.10	0.13	0.10
Femininity ideology	0.28	0.10	0.45	0.18*	0.23**	0.18
Masculinity ideology	0.36	0.11	0.62	0.16*	0.20**	0.16

*R*^2^ = 0.09, Adjusted *R*^2^ = 0.08, *F*(4, 284) = 7.30, *p* < 0.001.

*b*, Unstandardized coefficient; CI, Confidence interval; *ß*, Standardized coefficient; *r*, Zero-order correlation; *sr*^2^, Squared part correlation. *p < 0.02. **p < 0.001.

**Table 6:** Linear multiple regression predicting social media for appearance validation.

	*b*	CI_95%_ for b		*β*	*r*	*sr* ^2^
		Lower	Upper			
Predictor variables
Age	0.15	0.05	0.25	0.16*	0.13	0.16
Gender	0.53	0.33	0.73	0.28**	0.27**	0.28
Femininity ideology	0.19	0.03	0.34	0.13*	0.21**	0.13
Masculinity ideology	0.45	0.23	0.68	0.22**	0.26**	0.21

*R*^2^ = 0.18, Adjusted *R*^2^ = 0.17, *F*(4, 284) = 15.78, *p* < 0.001.

*b*, Unstandardized coefficient; CI, Confidence interval; *ß*, Standardized coefficient; *r*, Zero-order correlation; *sr*^2^, Squared part correlation. *p < 0.02. **p < 0.001.

For emotion bonding, the regression model was significant, accounting for 7% of the variance (Table 3). Gender (girls) and femininity ideology each uniquely contributed significantly to the model. Hypothesis 1B was partially supported—femininity ideology was uniquely predictive of emotion bonding but did not mediate the relationship between gender and emotion bonding.

For competitive activity bonding, the regression model was significant, accounting for 11% of the variance (Table 4). Gender (boys), masculinity ideology, and femininity ideology each uniquely contributed significantly to the model. Hypothesis 2B was partially supported—masculinity ideology was uniquely predictive of competitive activity bonding but did not mediate the relationship between gender and competitive activity bonding.

The model for social compensation was also significant, accounting for 8% of the variance (Table 5). Both masculinity and femininity ideologies uniquely contributed significantly to the model and the effect of gender was reduced to insignificance. Hypothesis 3B was partially supported - masculinity, but also femininity, ideologies were uniquely associated with social compensation and fully mediated the relationship between gender (girls) and social media use for social compensation.

For appearance validation, the regression model was significant, accounting for 17% of the variance (Table 6). Age, gender (girls), masculinity ideology, and femininity ideology each uniquely contributed significantly to the model. Hypothesis 4B was only partially supported; femininity ideology was uniquely associated with appearance validation but less so than masculinity ideology, and without mediating the relationship between gender and appearance validation.

Using Benjamini-Hochberg alpha correction procedure we rank ordered each of our *p* values for the 13 tests of hypotheses. Using this correction, the critical cutoff point was after rank order 10, *p* = 0.038. The largest value we identified as statistically significant was *p* = 0.02 (the unique association between femininity ideology and social media for appearance validation).

## Discussion

The aims of this study were to examine gender differences in the purposes of adolescents’ social media use and test whether identification with traditional masculinity and femininity ideologies explained those differences. We found that girls were more likely to report using social media for emotion bonding, appearance validation, and social compensation whereas boys were more likely to report using social media for competitive activity bonding. Girls and boys did not differ in their reports of social media use for bullying. They also did not differ in their degree of identification with dominant masculinity ideology, but girls identified more with submissive femininity ideology than boys, replicating recent research with these scales (e.g., Priess et al., 2009; Rogers et al., 2020). Regressions showed that gender ideologies contributed to adolescents’ social media purposes over and above gender identification but did not explain away gender differences (except for social compensation). Overall, masculinity ideology was most strongly associated with girls’ and boys’ social media purposes (except for emotion bonding, which was associated with femininity ideology).

### Masculinity ideology and adolescent social media use

Hegemonic masculinity operates in society and interpersonally through morphing strategies for gaining dominance and superiority in everyday social interactions (Connell and Messerschmidt, 2005), which may be magnified *via* social media affordances such as availability, publicness, permanence, and quantified social metrics (see Nesi et al., 2018). In addition, since the 1970s and so-called second wave feminist movement, there has been an increase in U.S. women’s identification with character traits such as assertiveness that have traditionally been associated with masculinity and men (Twenge, 2001). Developmental work has shown that in middle childhood, girls become more aware of power and status at the societal level and often respond by shifting from idealizing the “girly-girl” to striving to be “one of the boys” (Halim et al., 2011). By adolescence, girls tend to identify with masculinity ideology to a similar degree as boys (Priess et al., 2009; Rogers et al., 2020) and in adulthood, women’s and men’s identification with hegemonic masculinity predicts important outcomes such as evaluations of politicians accused of sexual assault (Schermerhorn et al., 2022). Our findings add to this body of work on the continued relevance of traditional masculinity ideology among all genders, suggesting that cultural beliefs and values for dominance and invulnerability in relationships can inform the needs adolescents seek to fulfill with social media.

Most studies on digitally-mediated friendships in adolescence focus on emotion bonding (e.g., Schouten et al., 2007), yet competitive activity bonding was the highest rated purpose of social media among all adolescents in our study and uniquely associated with identifying as a boy, and with masculinity and femininity ideologies. Online gaming tends not to be classified as social media use but has been shown to be conducive to social support and bonding (Trepte et al., 2012), although there is evidence that boys experience competitive peer relations as more supportive than do girls (De Goede et al., 2009; Hibbard and Buhrmester, 2010). Future studies should pay more attention to various ways adolescents’ bond online and consider how gender and gender ideologies may moderate socioemotional outcomes of competitive activities versus emotion bonding *via* social media. For example, boys and girls with greater identification with masculinity ideology in relationships may perceive gaming, debates, or one-upmanship on social media to be more fulfilling and conducive to friendship compared to those identifying less with masculinity ideology.

Values for dominance in relationships are often overlooked as a potential driver of girls’ social media use for appearance validation because girls’ desire to be sexually attractive to men has been theorized as a behavior resulting from girls’ internalization of submissive femininity ideologies (e.g., Tolman et al., 2006). Previous research has measured masculinity in boys and femininity in girls, showing that greater alignment between one’s gender identity and cultural ideologies for gender motivates self-objectification *via* social media (e.g., van Oosten et al., 2017). In contrast, our study indicates that for both girls and boys, hegemonic masculinity is a stronger influence than submissive femininity in the use of social media to post and receive attention for attractive photos and compare oneself to others’ appearances. This finding aligns with earlier observations of “raunch” culture (i.e., “girls gone wild”) and the ways young women engage in sexual self-objectification, not just to perform for men, but also to inhabit their sexual expression like men—assertively (Levy, 2005). Self-objectification and appearance validation may also be a way for adolescents to compete with friends. The tendency to position girls as victims of technology due to their insecurities, submissiveness, and desires to please others fails to recognize the complexity of girls’ motivations and how values for power, competition, status, and invulnerability inform this online behavior and who benefits from it (Renold and Ringrose, 2011). Interventions designed to address girls’ and boys’ social media use and body image as a source of depressive symptoms (e.g., Maheux et al., 2022) should also address beliefs and values long associated with dominant masculinity in motivating uses of social media for appearance validation and self-worth.

### Femininity ideology and adolescent social media use

The second most common purpose of social media use was emotion bonding with friends, which was higher among girls and uniquely predicted by femininity ideology, as expected. We hypothesized that cultural ideologies for gender would mediate the effects of gender identity on social media purposes. However, femininity ideology only added to the variability accounted for in emotion bonding. In other words, identification with submissive femininity ideology increased boys’ and girls’ social media use for emotion bonding, rather than explaining why the gender difference occurred. Few studies consider how boys identify with femininity ideology and the implications for their peer relations and socioemotional outcomes. Boys who identify with values for vulnerability in relationships may be more motivated to make use of communication affordances such as accessibility and reduced visual cues to express emotional openness and sensitivity with friends. We also urge caution in interpreting our findings related to femininity ideology, given the very low reliability of the scale (see limitations).

Social media for social compensation was the purpose with the smallest gender difference, which became insignificant in the regression model, suggesting full mediation through gender ideologies. Post-hoc regressions with femininity and masculinity in separate models showed that femininity was the ideology that accounted for the gender difference while masculinity ideology added additional variance. Social compensation has mostly been studied from the point of view of youth who are socially anxious and self-conscious in face-to-face contexts (e.g., Desjarlais and Willoughby, 2010) or in terms of individual differences in sociability and extraversion (Lee, 2009), thus less known about how beliefs and values regarding the self in relationships contribute to using social media for social compensation. The emphasis on vulnerability in femininity ideology could motivate a preference for computer-mediated communication *via* social media to increase intimacy beyond face-to-face communication while an emphasis on the self as independent in masculinity ideology could drive use of social media to seek new, looser relationships that promote individual mobility. Our operational definition of social compensation—the use of digital media to overcome limitations in face-to-face contexts— was too broad to examine this potential nuance. Social compensation may also look different among LGBTQ and gender non-binary youth who use social media to compensate for lack of community, information, and identity validation offline (Fox and Ralston, 2016). Further work on social media use for social compensation should consider defining more precisely the types of limitations that are occurring offline, the social resources that are gained online, and how limitations and resources are shaped by gender identity, as well as other positionalities such as race and class in society.

### Gender similarities in social media use

Despite our focus on gender differences, there were also many similarities in social media uses, supporting the gender similarities hypothesis (Hyde, 2014). The most common purpose of all adolescents’ social media use was to bond with friends through competitive activities, followed by emotion bonding, social compensation, appearance validation, and then bullying. There was also no gender difference in the use of social media for bullying. Further, Instagram is often associated with girls and selfies (Yau and Reich, 2018), but we found no associations between use of social media for appearance validation and this platform preference, perhaps because this platform was ubiquitous in our sample, among girls and boys. Indeed, most adolescents in our study reported using the same two platforms, Instagram and Snapchat. Although, there was a gender divergence in their third platform preference, which included relatively newer sites at the time such as TikTok (more popular with girls) and Discord (more popular with boys), which have now become popular among all adolescents.

### Limitations and future directions

Although the current study contributes to research and framing on gender and adolescent social media use, it is not without limitations. The sample in this study is mostly white and middle class, and exclusively cisgender; thus, findings should not be generalized beyond these identities. Our data are correlational and thus we cannot definitively say whether ideologies lead to social media uses or whether uses lead to ideologies. However, previous longitudinal research examining reciprocal relationships suggests adolescents’ gender role identity leads to certain forms of social media use, rather than the other way around (van Oosten et al., 2017). Longitudinal studies starting in middle to late childhood and following youth into adolescence and emerging adulthood would clarify how identification with gender ideologies change over time in relation to various forms of social media use.

Some of our measures did not meet the 0.70 cut-off for scale reliability, particularly our measures for femininity and masculinity ideologies. Due to school constraints for data collection, we used abbreviated versions of the scales, which may account for the low omega levels. Another possibility is that the scale items, constructed in the early 2000’s, are less relevant to contemporary youth and do not hang together as clear constructs. This is a likely possibility given that traditional gender role attitudes valuing power for men and submissiveness for women have been in decline in the U.S. since the 1970s (Twenge, 1997). It is notable that our factor analysis showed the improvement of the femininity ideology scale with the removal of items related to self-expression, suggesting that self-silencing may be less relevant to performing submissive femininity compared to the past, before social media. In addition, our study was limited to traditional European-American gender ideologies and did not consider ideologies that might be more relevant to ethnic or racial minority youths’ social media use, such as the Strong Black Woman ideology (e.g., Anyiwo et al., 2018).

Prior work using a UGT framework to examine social media use motivations has documented specific reasons for using a particular platform (e.g., Phua et al., 2017) but we sought to examine common purposes relevant to adolescents’ idiosyncratic polymedia environments. Thus, while this research breaks new ground in conceptualizing social media use beyond simple frequency of use, we acknowledge that we have not covered the full scope of reasons why adolescents use social media in their polymedia environments. Although most of our social media purposes scales worked well, we recommend improvements to the social compensation scale, as described above, and recommend strengthening the competitive activity scale items so that they more clearly capture the intended construct—a combination of competitiveness with fun and connection, which may require more scale items.

### Conclusions and contributions

Adolescents in contemporary polymedia environments are customizing their experiences according to cultural ideals and personal needs such that evaluations of the effects of social media on adolescent well-being make little sense outside the personal and cultural contexts in which they occur (Odgers, 2018; Manago and McKenzie, 2022). Overall, our findings demonstrate that gender remains an important context for understanding individual differences in motivations for social media use among U.S. adolescents. Similar to previous research showing that gender ideologies mediate motivations and access to opportunities for learning and development in complex, sometimes contradictory, ways (Sung, 2021), our study suggests that gender binary frameworks presuming distinct ideologies leading to divergent purposes among girls and boys are not complex enough for understanding how social constructions of gender influence social media use among contemporary adolescents. Clarifying how gender ideologies are shifting, not only among LGBTQ+ youth but also cisgender and heterosexual youth, will be key for understanding gender as a cultural context for adolescents’ social media use and for addressing digital inequities in modern technological societies that have evolved from first-level divides about access, to new kinds of disparities in how digital tools are used and who benefits from them (Ragnedda, 2017; Scheerder et al., 2017).

## Data availability statement

The raw data supporting the conclusions of this article will be made available by the authors, without undue reservation.

## Ethics statement

The studies involving human participants were reviewed and approved by UC Santa Cruz Institutional Review Board #3375 “Gender and Sexual Identity Development on Social Media.” Written informed consent to participate in this study was provided by the participants’ legal guardian/next of kin informed consent was provided by participants and their legal guardian/parent.

## Author contributions

The first author AM led the design, data analysis, conceptualization, and writing of the paper. The second author AW contributed to the design, conceptualization, and writing of the paper. The third author LB contributed to the conceptualization and writing of the paper. All authors contributed to the article and approved the submitted version.

## Funding

The research was supported by a Hellman Fellowship and the Institute for Social Transformation, both housed at the University of California, Santa Cruz.

## Conflict of interest

The authors declare that the research was conducted in the absence of any commercial or financial relationships that could be construed as a potential conflict of interest.

## Publisher’s note

All claims expressed in this article are solely those of the authors and do not necessarily represent those of their affiliated organizations, or those of the publisher, the editors and the reviewers. Any product that may be evaluated in this article, or claim that may be made by its manufacturer, is not guaranteed or endorsed by the publisher.

## Supplementary material

The Supplementary material for this article can be found online at: https://www.frontiersin.org/articles/10.3389/fpsyg.2022.1011951/full#supplementary-material
