# Supplementary material for: The contributions of gender identification and gender ideologies to the purposes of social media use in adolescence
Source: Front Psychol. 2023 Jan 10;13:1011951. doi: 10.3389/fpsyg.2022.1011951 (PMC9871900; doi:10.3389/fpsyg.2022.1011951)
Supplement: Supplementary file 1 [file Table_1.pdf]

Appendix

Results From a Factor Analysis of the Femininity Ideology Scale

Femininity Ideology Scale	Factor loading	
	1	2
I express my opinions only if I can think of a nice way of doing it.	.20	-.66
I worry that I would make others feel bad if I am successful	.67	-.23
I often change the way I do things in order to please someone else.	.76	-.07
I tell my friends what I think even when it is an unpopular idea	.22	.65
Often, I look happy on the outside in order to please others, even if I don't feel happy on the inside	.71	-.08
I usually tell my friends when they hurt my feelings.	.39	.65

Note. N = 305. The extraction method was principal axis factoring.

Results From a Factor Analysis of the Masculinity Ideology Scale

Masculinity Ideology Scale	Factor loading	
	1	2
In a good dating relationship, I would get my way most of the time.	.52	.15
Sometimes I feel like if I hit someone, I would get more respect.	.58	.44
Telling people my worries would make me look weak.	.65	-.56
It's important to go after what I want, even if it means hurting other people's feelings	.63	.29
It's important to act like I am sexually active and knowledgeable, even if I am not.	.51	.41
It's embarrassing to me to ask for help.	.54	-.67

Note. N = 302. The extraction method was principal axis factoring.

Results From a Factor Analysis of the Social Media Purposes

Social Media Purposes	Factor Loading				
	1	2	3	4	5
I use social media to share personal thoughts and feelings with close friends.	.67	-.32	.24	-.20	-.25
I use social media to talk about personal problems with close friends.	.64	-.37	.23	-.20	-.40
I use social media to show emotional support to my close friends.	.59	-.43	.28	-.13	-.29
I use social media to challenge my close friends to see who is better at something.	.48	.31	.29	-.10	.09
I use social media to joke around with my close friends.	.47	-.12	.46	-.17	.50
I use social media to engage in fun activities with my close friends.	.54	-.14	.33	.01	.54
I use social media to see what others think about how I look.	.63	.01	-.63	-.18	.03
I use social media to see how my appearance compares to others.	.60	-.04	-.62	-.22	.03
I use social media to show "hot" photos of myself.	.50	.11	-.48	-.33	.21
I use social media to find new friends.	.57	-.16	-.17	.53	.23
I use social media to talk about things that I don't want to talk about face-to-face.	.59	-.18	.06	.38	-.15
I use social media to interact with people who are more like me than the people I know from school.	.44	-.12	-.16	.71	-.01
I threatened to hit, kick, push, or shove a peer on social media.	.35	.77	.19	.04	.01
I called peers mean names or teased them in a hurtful way on social media.	.38	.73	.10	-.04	.08
I have engaged in "drama" online (interpersonal conflict with friends on social media).	.47	.45	-.01	.03	-.22
I spread false rumors about peers on social media and tried to make others dislike them.	.23	.61	.10	.12	-.38

Note. $N = 305$. The extraction method was principal axis factoring with an oblique (Promax with Kaiser Normalization) rotation.
